# The effect of ionic association on the electrochemistry of redox mediators for Li–O_2_ batteries: developing a theoretical framework†

**DOI:** 10.1039/d4cp01488j

**Published:** 2024-07-01

**Authors:** Gabriela Horwitz, Vera Kunz, Samuel P. Niblett, Clare P. Grey

**Affiliations:** a Yusuf Hamied Department of Chemistry, University of Cambridge, Lensfield Rd Cambridge CB2 1EW UK cpg27@cam.ac.uk; b The Faraday Institution, Quad One, Harwell Campus, Becquerel Ave Didcot OX11 0RA UK

## Abstract

A theoretical framework to explain how interactions between redox mediators (RMs) and electrolyte components impact electron transfer kinetics, thermodynamics, and catalytic efficiency is presented. Specifically focusing on ionic association, 2,5-di-*tert*-butyl-1,4-benzoquinone (DBBQ) is used as a case study to demonstrate these effects. Our analytical equations reveal how the observed redox couple's potential and electron transfer rate constants evolve with Li^+^ concentration, resulting from different redox activity mechanisms. Experimental validation by cyclic voltammetry measurements shows that DBBQ binds to three Li^+^ ions in its reduced state and one Li^+^ ion in its neutral form, leading to a maximum in the electron transfer kinetic constant at around 0.25 M. The framework is extended to account for other phenomena that can play an important role in the redox reaction mechanisms of RMs. The effect of Li^+^ ion solvation and its association with the supporting salt counteranion on the redox processes is considered, and the role of “free Li^+^” concentration in determining the electrochemical behaviour is emphasized. The impact of Li^+^ concentration on oxygen reduction reaction (ORR) catalysis was then explored, again using DBBQ and modelling the effects of the Li^+^ concentration on electron transfer and catalytic kinetics. We show that even though the observed catalytic rate constant increases with Li^+^ concentration, the overall catalysis can become more sluggish depending on the electron transfer pathway. Cyclic voltammograms are presented as illustrative examples. The strength of the proposed theoretical framework lies in its adaptability to a wider range of redox mediators and their interactions with the various electrolyte components and redox active molecules such as oxygen. By understanding these effects, we open up new avenues to tune electron transfer and catalytic kinetics and thus improve the energy efficiency and rate capability of Li–O_2_ batteries. Although exact results may not transfer to different solvents, the predictions of our model will provide a starting point for future studies of similar systems, and the model itself is easily extensible to new chemistries.

## Abstract 2

The kinetics and thermodynamics of the electrochemical reactions of redox mediators for lithium–air batteries depend on ionic association strength with Li^+^ ions and on specific pathways, potentially affecting the energetic efficiency of devices.

## Graphical abstract

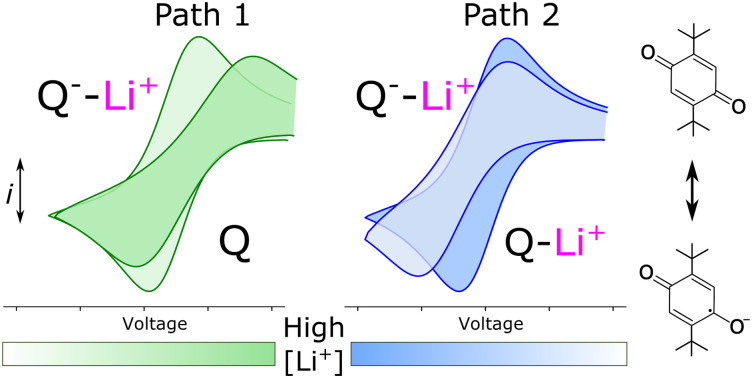

## Introduction

Lithium–air batteries (LABs) have a theoretical energy storage capacity three to five times higher than Li-ion batteries and are thus appealing candidates for high-energy density electric vehicle applications. They are composed of a lithium anode and a porous conductive matrix (usually carbon-based), at which oxygen is reduced during discharge and the discharge product, typically lithium peroxide, is oxidized during charge. One of the major issues preventing the development of LABs is the high overpotential needed to charge the battery due to the insulating nature of the solid discharge products, leading to a cascade of degradation reactions.^1,2^

One strategy to help enable the discharge and charge reactions of the LAB, is to use soluble redox-active species (redox mediators, RMs) to increase energy efficiency and minimize electrolyte degradation. The use of these soluble species is a widespread strategy across different applications in electrochemical systems.^3^ Some examples include redox-flow batteries, where the dissolved electroactive species are the main source of energy storage, and hybrid solid redox flow batteries, where RMs transport the charge between an electrode and a disconnected solid active species.^4^ A similar concept, “redox targeting” was also introduced to overcome the insulating nature of lithium insertion materials (LiFePO_4_).^5^ Furthermore, soluble active species have been used as a liquid anode in alkali metal batteries (Na and K) coupled with different cathodes, including oxygen.^6^

When RMs are present in a LAB, these molecules or ions act as soluble catalysts: during the discharge, the RM is reduced at the surface of the electrode; it then diffuses through the solution where it can react with dissolved oxygen, eventually forming Li_2_O_2_ as the discharge product. As the crystallization of lithium peroxide occurs from the solution, large Li_2_O_2_ crystals can be formed while leaving free, non-passivated electrochemically active surface, leading to high discharge capacities. During charging, the RM is oxidized at the exposed, free surface of the electrode that is not blocked by discharge product, and then chemically reacts with it to release O_2_. Thus, RMs allow decoupling of the oxygen reduction reaction (ORR) and oxygen evolution reaction (OER) from the electron transfer (ET) reactions at the electrode, giving the charge transfer an alternative path to tunnelling through the electrically insulating Li_2_O_2_.^1^ Furthermore, the use of RMs can avoid reaction pathways that result in reactive oxygen species and hence improve the overall cycling stability of LABs.^7–10^ One of the most commonly used discharge RMs is 2,5-di-*tert*-butyl-1,4-benzoquinone (DBBQ), which has been shown to increase the discharge capacity of a Li–O_2_ battery up to 80 times under typical LAB operating conditions.^11^

Previous studies have mainly focused on understanding the thermodynamic aspects governing the ability of a molecule to act as a successful RM. The principal parameter analysed is usually the standard potential of the RM electrochemical couple, *E*^0^, although this quantity is usually obtained using a Li reference electrode, which is potentially complex since the electrode is reactive and not always stable and the Li/Li^+^ potential varies between different electrolytes.^12,13^ Moreover, the *E*^0^ of the RMs is also often incorrectly assumed to be independent of the solvent, Li salt identity and concentration. Some attention has been given to the effect of solvation environments on the reduction potential of iodide,^14^ but little has been said about other RMs.^15^ Since most ET reactions with RMs involve a significant change in the charge/size ratio of the molecule or ion, it is only natural that the driving force for this reaction will be dependent on the molecular environment around the species.

The kinetics of the ET and of the reaction of RM with the target species (O_2_ in the case of discharge RM and Li_2_O_2_ in the case of charge) are another factor that have received almost no attention, and yet they are crucial for the operation of a Li–O_2_ battery. The kinetics of ET directly controls the overpotential in a galvanostatic battery configuration, while the rate of catalysis needs to be fast enough to sustain the cycling. It is often assumed that the electrochemical conversion of RMs is fast, and hence Nernstian. However, most RMs exhibit quasireversible behaviour, which can limit the viable current density.^16–18^ In fact, significant overpotentials are seen when discharging Li–O_2_ batteries even when using RMs under high rates that are closer to practical values. Some effort has been made to relate the kinetics of the catalysis to the standard potential of the charge RM or the ET kinetics, but there is no consensus regarding these effects.^19–23^ Recently, Bawol *et al.* showed the importance of ionic association in the kinetics of ORR catalysis by DBBQ,^15^ proposing that a complex of Li-DBBQ mediates the reaction and showing that a higher association of DBBQ with a cation promotes faster catalysis.

The aim of this work is to establish relationships between chemical parameters, such as ionic association, solvation energy and parallel equilibria, and the driving force and kinetics of ET involving the RM, and hence, to understand the RM's catalytic role. These effects are directly related to the performance of a Li–O_2_ battery. We use DBBQ in Li^+^-containing dimethylsulfoxide (DMSO) solutions as a model electrolyte to illustrate the approach, because of its importance for Li–O_2_ batteries, however the approach presented here can be easily applied to other redox active species, or other discharge and charge RMs, in solvents where ionic pairing is expected to be significant.

DBBQ is known to undergo ionic association with up to three lithium ions, as seen from the dependence of its reduction potential on the concentration of lithium salt in DMSO electrolytes.^15^ The exact mechanism for this reduction has been sometimes assumed to be either concerted (eqn (1)),^16,24^ or stepwise (eqn (2)).^15^

1 DBBQ + *n*Li + e^−^ ⇄ DBBQ⋯Li_*n*_

2
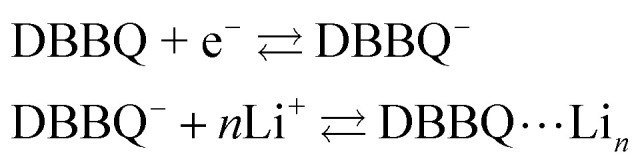


Inspired by the well-established behaviour of quinones in aqueous conditions, which undergo proton-coupled electron transfer, this work first develops a theoretical framework to treat RM's ionic association with Li^+^ ions based on the Nernst and the Butler–Volmer (BV) equations. Our framework is written in terms of a general RM, denoted as Q, which is assumed in the equations to be neutral in its initial state; however, equivalent equations can be written for the case of charged RMs.

We start by analysing how association constants and Li^+^ concentration impact the thermodynamics and kinetics of different possible pathways, defining an apparent rate constant and showing how it changes as a function of [Li^+^]. By comparing the framework predictions with experimental data obtained by cyclic voltammetry, we extract insight into the mechanism of DBBQ conversion in DMSO based electrolytes. To expand the framework's validity into systems composed of different solvents and salts, we then explore the theoretical consequences of other phenomena, namely counteranion and solvation effects. Since a typical electrolyte for Li–air batteries is composed of a lithium salt (sometimes referred to as a “supporting salt”) and a RM dissolved in much lower concentrations, we analyse the effect of competition for Li^+^ ions with the lithium salt counteranion, giving one more dimension to the tuning of these processes. Given further experimental data, this analysis will help determine the mechanisms by which DBBQ operates in various non-aqueous electrolytes at concentrations relevant for batteries. Furthermore, our approach opens a new route to rationally tune ET kinetics for RMs by optimizing electrolyte composition for increased electrochemical rates and hence lower overpotentials.

Once the effect of ionic association in the ET of RMs is understood, we move on to analyse the effect of ion association on the oxygen reduction reaction (ORR) catalysis. In the case of DBBQ, it has been established that the reactive intermediate is an associated monoanion-Li (DBBQ–Li_*n*_).^15^

The elementary steps of DBBQ's reactions have been proposed to start with the association of the reduced quinone to O_2_, which is expected to be reversible^15,16,25^3
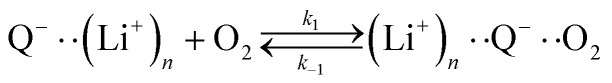
This complex can then disproportionate following different paths leading directly to the final product Li_2_O_2_ or the intermediate LiO_2_. Proposed mechanisms are shown in eqn (4)–(6). The complex can disproportionate directly:4
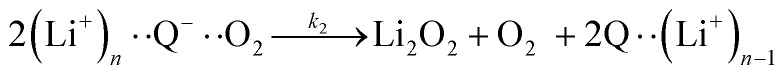
or it can react with another reduced (Li^+^-associated) quinone:5
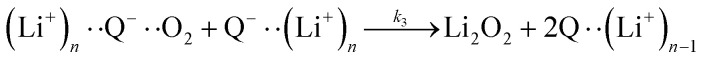
Or finally, it can disproportionate by a unimolecular reaction to produce lithium superoxide and (Li^+^-associated) oxidized quinone, and the lithium superoxide then can disproportionate into oxygen and lithium peroxide.6a
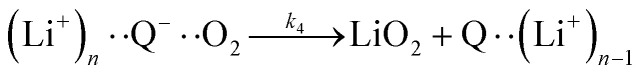
6b
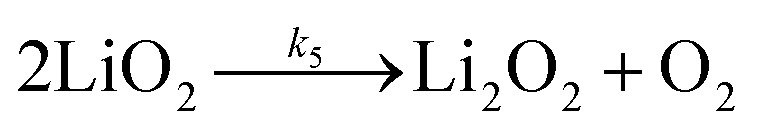


It is worth noting that reaction (4) is a combination of both steps in reaction (6). To maintain consistency with previous reports,^15^ we have named the rate constants in eqn (3) and (6)*k*_1_, *k*_−1_, *k*_4_ and *k*_5_. Bawol *et al.* have found increased catalytic rates in solutions with higher ionic association of DBBQ, indicating that associated DBBQ species are the active ones during ORR.^15^ Here, we have considered the possibility that not only DBBQ–Li^+^ takes part in the reaction, but highly associated quinone species (*n* ≥ 1) can also be involved.

This work ends by establishing a theoretical simplified representation of how association strength affects the ORR kinetics and cyclic voltammograms. This will allow the changes in in electrochemical signatures related to association and solvation environments to be explained. Therefore, the predictions made here will serve as a guide to extract qualitative (or even quantitative) mechanistic information from the results of these types of electrochemical experiments.

## Results and discussion

### 1. Theoretical framework: effect of ionic association on different electron transfer mechanisms under an inert atmosphere

In this section, we present a general model to describe how the thermodynamic and kinetic parameters of a one-electron reaction are affected by the ionic association of the redox-active species with another ionic species using DBBQ as the model RM. For simplicity, we first assume an inert atmosphere (*e.g.* Ar gas) so there is no oxygen gas available to react with any component of the electrolyte. We show how this model can be used to diagnose specific pathways the redox reaction can take, helping to understand the differences observed under various experimental conditions; moreover, we show how we can use the model as a tool to tune the desired path.

**Scheme 1:**
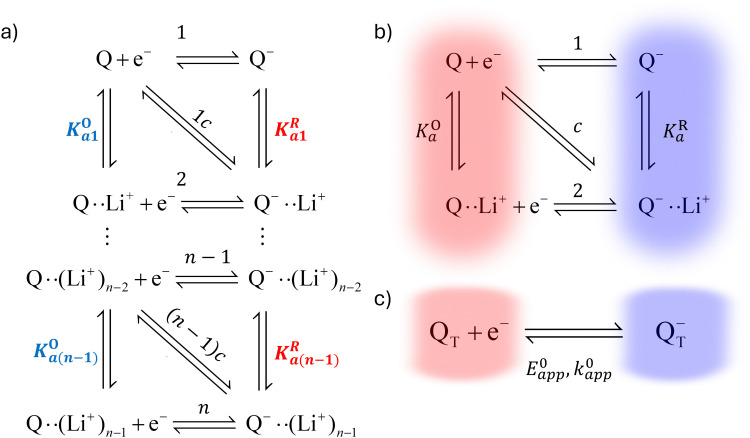
Possible reactions in non-aqueous lithium-salt-containing electrolytes for a general RM, Q. Panel (a) is generalized to multiple Li^+^ interactions and (b) only considers interactions with one Li^+^ ion. The monomolecular/monoionic (horizontal) ET reactions are numbered from 1 to *n*, and the bimolecular steps (diagonal) are numbered 1c to (*n* − 1)c, “c” indicating that the reaction is concerted. (c) shows a simplified mechanism where the species shaded red in panel (b) are combined into an effective species Q_T_, and the ones shaded in blue are combined into Q_T_^−^.

It is known that the DBBQ monoanion bonds to multiple lithium ions in DMSO-based electrolytes. Here, we allow for the possibility that the oxidized neutral DBBQ also bonds to Li^+^ ions. Taking this into account, the first reduction of DBBQ can, in principle, follow multiple pathways, depicted in Scheme 1a, where the vertical reactions represent chemical equilibria of association of quinone species with Li^+^ ions, the horizontal ones represent ET reactions, and the diagonal ones concerted steps where association and ET happen at the same time. To simplify the theoretical treatment, we first consider the uppermost square of the ladder, depicted in Scheme 1b, before extending to the more general case. For simplicity, and given that this treatment is general, we will call our reactive species Q.

#### 1.1 Thermodynamic considerations

First, we consider three possible mechanisms for the redox activity of the first reduction of Q (Scheme 1b). Q can be reduced in its free form, while bound to a lithium ion, or in a concerted step. Following the Butler–Volmer theory of electron transfer, we define *E*^0^_i_, *k*^0^_i_ and *α*_i_ as the standard potential, standard rate constant, and transfer coefficient of each of the possible *n* elemental steps.^26^*α*_i_ corresponds to the symmetry of the potential barrier, which we assume is the same for all reactions considered in this paper, in accordance with the literature on proton-coupled electron transfer reactions.^27^

In addition to the elemental electrochemical steps (horizontal reactions), we consider the simultaneous equilibrium of each of the associated species with its dissociated form (vertical reactions). These reactions are governed by *K*^O^_a_ and *K*^R^_a_, the association constants of each of the species with the complexing ion present in solution (Li^+^ in this example). The RM in our work, Q, is assumed to be neutral, so the Q–Li^+^ coulombic interactions will be smaller than those for Q^−^–Li^+^. Therefore we assume *K*^O^_a_ to be smaller than *K*^R^_a_. However, the RM could be initially charged (a reduction of Q^−^ to Q^2−^, for example) and our framework would still be applicable. These reactions can be summarized in a square type diagram as represented in Scheme 1, which resembles analogous mechanisms proposed in proton-coupled redox reactions.^27–29^ Which reduction pathway DBBQ undergoes will depend on the key parameters: the lithium concentration, *K*^O^_a_, *K*^R^_a_, *E*^0^_1_ and *E*^0^_2_ the standard potentials of the elemental steps denoted as 1 and 2 in Scheme 1b.

The relations between the standard potential of the three different electrochemical reactions are defined by the strength of ionic association and can be described by eqn (7) and (8) coming simply form the relation between Δ*G*^0^, Δ*E*^0^ and *K*^i^_a_.7
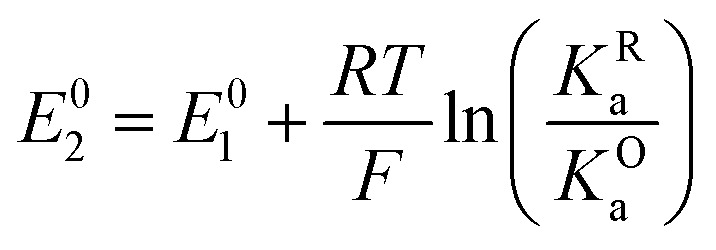
8
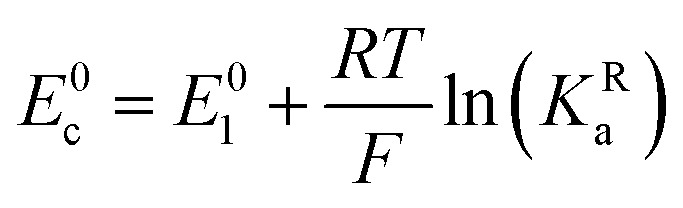


Taking inspiration from the proton coupled electron transfer literature and some previous ionic association studies,^27,28,30–32^ we define the overall apparent standard potential, *E*^0^_app_, as the potential in which the total oxidized and reduced species reach the half-point, *i.e*, [Q^−^] + [QLi] = [Q] + [QLi^+^]. Given the simultaneous association equilibria, this potential will depend on lithium concentration. The rate at which the overall reaction takes place will also depend on lithium concentration and can be described by the apparent standard heterogeneous electron transfer rate constants, *k*^0^_app_, which will be discussed in more detail in the next section. It is important to note that these apparent parameters are the ones that can be directly measured experimentally, for example by the use of cyclic voltammetry.

The mass balance for Q at a particular potential *E* can be described by eqn (9) where [i] denotes the concentration of the species *i* in solution and *c*_*i*_ = [i]_T_ the total analytical concentration dependent on the potential through the Nernst equation.9
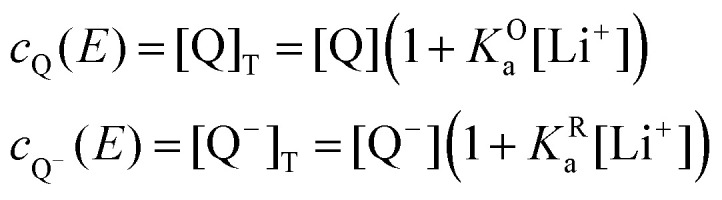
And the same can be done for the supporting salt, which exists either in associated form LiX or as free Li^+^ and X^−^ ions:10
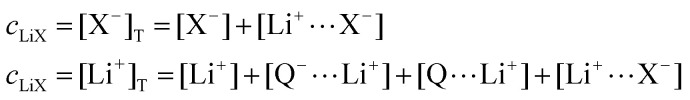
We first consider the simplest case where the free Li^+^ concentration is much higher than that of the RM, and its ionic association to the supporting salt's counteranion is negligible. In this case, eqn (10) become11
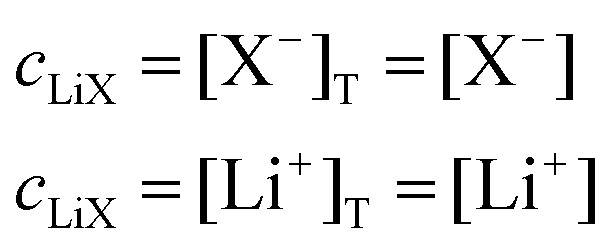
12
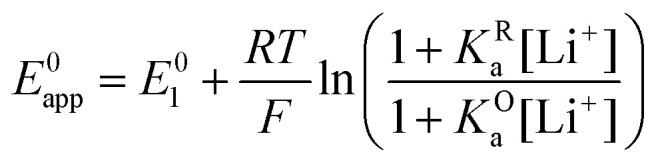
and the apparent potential of the overall system is given by eqn (12).

**Fig. 1:**
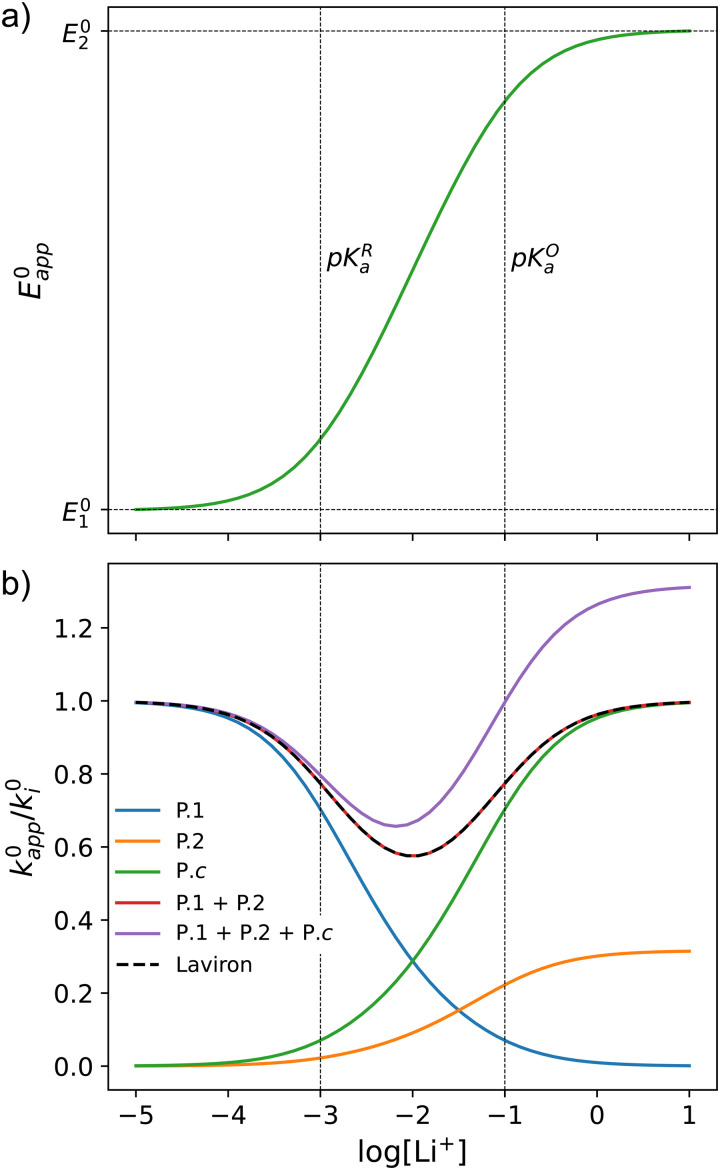
(a) Evolution of apparent potential of the system as a function of lithium concentration. (b) Evolution of individual components of the normalized rate constant and total rate constant as a function of lithium concentration. P.1, P.2, and P.*c* represent pathways 1, 2 and *c*, respectively. The black dashed line corresponds to the expression obtained by Laviron^28^ for the total kinetic constant, considering pathways 1 and 2, *k*^0^_1,app_ + *k*^0^_2,app_ (see text in Section 1.2 for definition), assuming *α*_1_ = *α*_2_ = 0.5. Figures were computed using *K*^O^_a_ = 10, *K*^R^_a_ = 1000, *k*^0^_1_ = *k*^0^_2_ = *k*^0^_3_ = 0.01.

This equation describes an “S” shaped curve, analogous to a Pourbaix diagram when dealing with protonation reactions, as shown in Fig. 1. The diagram is composed of three main regions: two constant-potential regions where the lithium concentration is much higher than 1/*K*^O^_a_, or much lower than 1/*K*^R^_a_, and a close to linear evolution between those values. These regions reflect a transition from pathway 1 to pathway 2 at different lithium concentrations if *K*^O^_a_ and *K*^R^_a_ are non-negligible.

It is important to note that in eqn (12), [Li^+^] represents the concentration of free lithium ions in solution (as opposed to their analytical concentration, which can be different in the presence of other equilibria). We assume that there are no reactions that might consume Li^+^, for the remainder of the current section, but later relax the assumption.

**Scheme 2:**
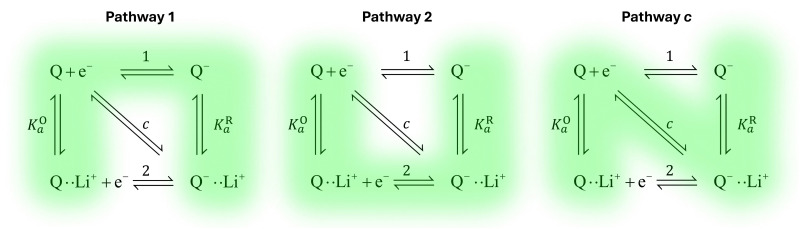
Representation of pathways 1, 2 and *c*, the green shading highlighting all the reactions that take part on the different pathways. For example, in pathway 1, the electron transfer reaction occurs *via* step 1, but the chemical equilibria involving Li^+^ association *K*^O/R^_1_ are also included in the modelling. Each of these pathways represent a subset of the ones presented in Scheme 1.

Extensive theoretical work has been performed by Laviron regarding coupled and closed sets of chemical and electrochemical reactions that can be depicted by a square, with an emphasis on protonation reactions.^27,28^ These systems are referred to here (and in the literature) as “square schemes”. In his seminal work, Laviron obtained expressions for the evolution of *E*^0^ and *k*^0^ as a function of the pH and p*K*_a_ of weak acids in schemes analogous to Scheme 1b, where instead of Li^+^ he considered H^+^ and a concerted mechanism was not taken into account. In his calculations, an *α* value of 0.5 for both horizontal ET reactions was assumed, resulting in an overall *k*^0^_app_ expression that can be interrogated to obtain reaction pathway ratios. For simplicity, and to obtain direct information about the different mechanisms, here we treat each pathway in Scheme 2 separately, before combining them to obtain the overall behaviour.

By combining eqn (7)–(9), alternative expressions for *E*^0^_app_ can be written, which will be useful in the next section when analysing each mechanistic scenario. Note that each of the eqn (12)–(14) depends on a different *E*^0^_i_, which means that they reflect how *E*^0^_app_ evolves when compared to the standard reduction potential of the ET steps involved in the different pathways.13
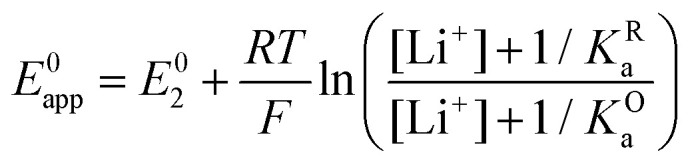
14
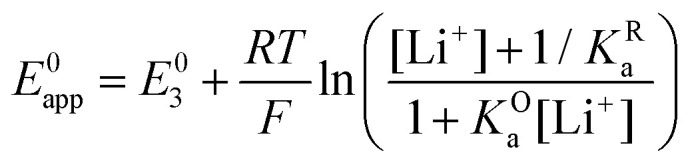


#### 1.2 Kinetic considerations

The first scenario we analyse is the one where the unbound species Q undergoes the electron transfer, denoted as pathway 1 in Scheme 2. One would expect this mechanism to dominate if the association of the oxidized neutral Q with lithium ions (red/orange equilibrium in Schemes 1 and 2) is negligible, either because *K*^O^_a_ is very small (expected in relatively high-dielectric constant solvents) or because [Li^+^] is low. However, since quinone association constants have been reported to be very high even in high dielectric solvents such as DMSO,^30^ and to keep the treatment general, we include the equilibrium Q + Li^+^ ⇌ QLi^+^ in the following analysis.

The rate of the reduction and oxidation processes for the elemental step 1, *v*^red^_1_, is described by the change in concentration of Q with time through pathway 1. Here, we only include the theoretical treatment for the reduction reaction, however, the same results can be obtained if the equations are expressed for the oxidation reaction instead.15
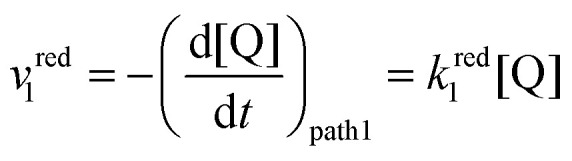
As it describes an elementary electrochemical process, we assume the rate constant *k*^red^_1_ follows the BV equation16
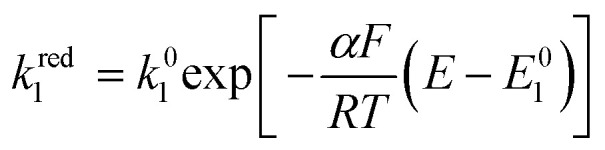
where *k*^red^_1_ is the first order heterogeneous rate constant at the potential *E*, and *k*^0^_1_ is the standard electron transfer rate constant for step 1.

If we combine eqn (12), (15) and (16) so that the rate of the reduction reaction is expressed as a function of *E*^0^_app_, the overall rate is defined by eqn (17).17
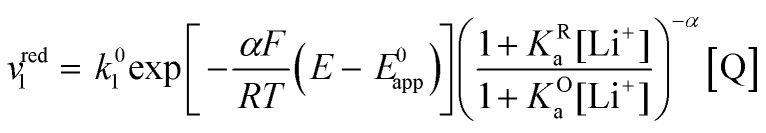
Using the fact that [Q] = [Q]_T_/(1 + *K*^O^_a_[Li^+^]) from eqn (9), we can write18
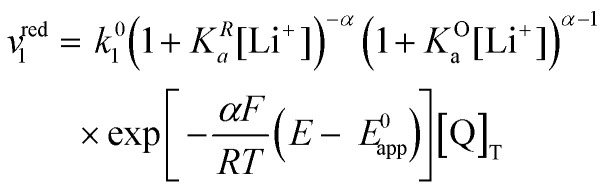
and it becomes evident that the process behaves as a single electrochemical couple with redox active species concentration [Q]_T_, standard reduction potential *E*^0^_app_, and standard electron transfer constant, *k*^0^_1,app_. This last quantity is defined as:19
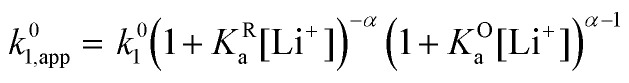


Now let's turn to pathway 2. In this case, only the lower electrochemical reaction in Scheme 1b is active, which is expected to be relevant at high lithium concentrations. The rate for reduction along this path is given by eqn (20). Analogously to pathway 1, if we replace *E*^0^_2_ by its expression as a function of *E*^0^_app_ from eqn (13), we can show that the system behaves as a single electrochemical couple with standard potential *E*^0^_app_, standard heterogeneous rate constant *k*^0^_2,app_ given by eqn (21), and redox active species concentration [Q]_T_.20
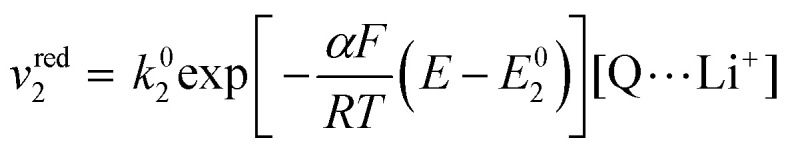
21
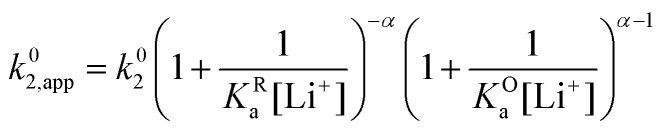
The third possible pathway that the reaction can take is the one where no intermediate is formed, *i.e.*, the reduction from the unbound quinone to the bound semiquinone happens in a concerted manner. In this mechanism, we can express the rate of the forward reaction, *v*^red^_c_, now as a function of the second order rate constant *k*^0^_c_ and the standard potential *E*^0^_c_.22
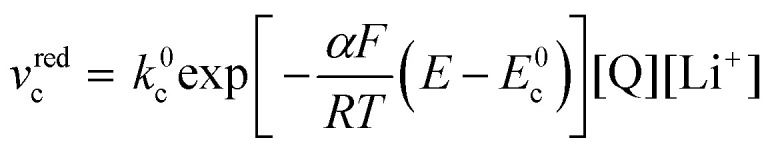
Analogous to the cases of pathways 1 and 2, one can obtain a new apparent standard electron transfer rate constant by replacing *E*^0^_c_ by its equivalent as a function of *E*^0^_app_, and [Q] by [Q]_T_. In this case, we have expressed the new apparent rate constant as a pseudo first order rate law, denoting this with the prime in 
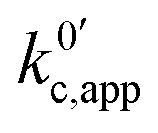
.23
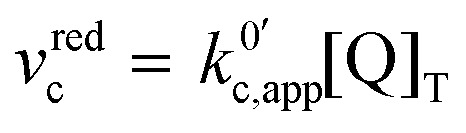
24
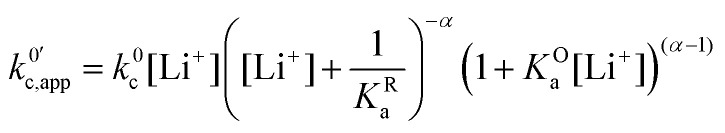

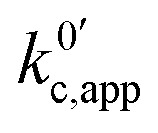
 now includes the [Li^+^] present in the rate law described in eqn (22). This comes with the approximation that the [Li^+^] stays constant throughout the reaction, without the need to introduce any new assumptions (since we already stated that [Q] ≪ [Li^+^]). In the extreme scenario, where the intermediate species Q^−^ and QLi^+^ are both highly energetic, and this is the only taken pathway, *i.e. K*^O^_a_ is extremely small and *K*^R^_a_ extremely large, we recover the expression obtained by Saveant for the purely concerted reaction.^33^25
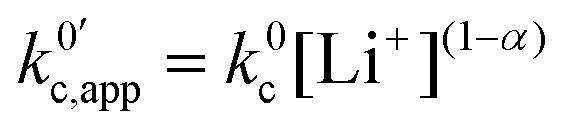


#### 1.3 Competition between the different pathways

We have shown that under certain common approximations, each of the pathways behaves as a single electrochemical couple with respect to the total amount of oxidized and reduced species, regardless of their degree of ion pairing. The whole system behaves the same way, with relative contributions from the different pathways varying as a function of lithium concentration. The total apparent standard rate constant, *k*^0^_app,T_, is given by (see deduction in the ESI,† eqn (S1)):26
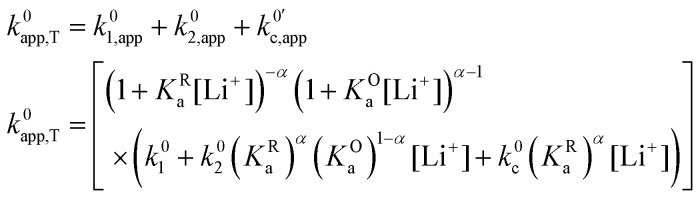


As expected, if we only consider pathways 1 and 2, and we assume that *α*_1_ = *α*_2_ = 0.5, then by adding up the resulting *k*^0^_1,app_ and *k*^0^_2,app_ we recover the expression obtained by Laviron for the total kinetic constant.^27,28^Fig. 1b nicely illustrates how the rates of pathway 1 and pathway 2 evolve with lithium concentration, and how at intermediate concentrations the reaction is more sluggish than either of the individual components at lower and higher [Li^+^]. If we now add the possibility of the reaction occurring partially though pathway c, we obtain the purple curve in Fig. 1b. This approach clearly represents the fraction of the reaction going through each of the pathways, proportional to the individual rate constants represented by *k*^0^_1,app_ and *k*^0^_2,app_ and 
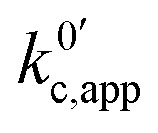
. It also depicts very clearly how, for a set value of *K*^O^_a_ and *K*^R^_a_, tweaking the concentration can promote either pathway 1 or pathway 2 or c. The competition between pathways 2 and c is largely determined by whether *K*^O^_a_ is higher or lower than 1, which reflects the relative stability of the intermediate Q⋯Li^+^. However, we note that the complex concerted mechanism necessary for pathway c could result in a low kinetic prefactor *k*^0^_c_, such that this pathway is not observed even in cases where the alternative pathways are thermodynamically disfavoured.

**Fig. 2:**
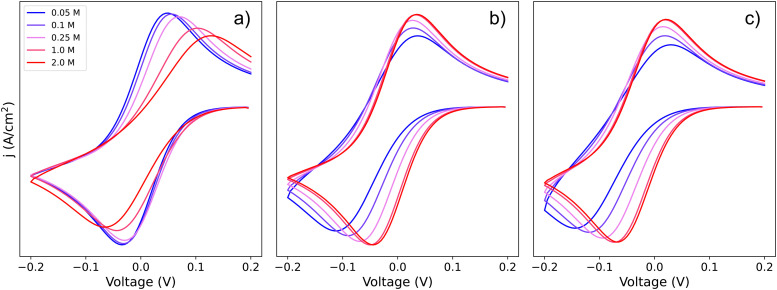
Simulated cyclic voltammograms for each of the pathways depicted in Scheme 2: pathways, (a) 1, (b) 2, and (c) c. The three plots are computed using *K*^O^_a_ = 2, *K*^R^_a_ = 10 and *k*^0^_1_ = *k*^0^_2_ = *k*^0^_3_ = 0.01, all with a scan rate of 50 mV s^−1^ and [Q] = 5 mM, for [Li^+^] ranging from 0.05–2 M.

To visualize how each of the pathways will manifest themselves in the experimental cyclic voltammogram (CV) data, Fig. 2 shows CVs of the system simulated using a finite element approach by DigiElch,^34^ which uses a numerical solution of the electrochemical system including both kinetics and mass transport. Each of the panels represents the system when it is allowed to take only one of the three pathways shown in Scheme 2. In the simulations, each elementary step was included, and CV response was obtained. The resulting CVs showed a single peak, and the effective parameters, *E*_app_ and *k*^0^_app_, of Scheme 1c were adjusted to reproduce this signal. These values were compared with the ones predicted by our framework in eqn (19), (21) and (24) and are in quantitative agreement within the limits of the approximations (*i.e.* [Li^+^] much higher than [Q]_T_). This confirms the validity of our framework when including mass transport effects.

If we focus on Fig. 2a, representing pathway 1, we can see that the peak-to-peak separation, a proxy for the rate of electron transfer,^26^ increases with Li^+^ concentration. This is in agreement with the predicted decrease in *k*^0^_1,app_ for this pathway with increasing [Li^+^]. For pathways 2 and c, the opposite trend appears: the peak to peak separation decreases with Li^+^ concentration, in agreement with the prediction seen in eqn (21) and (24) where *k*^0^_2,app_ and 
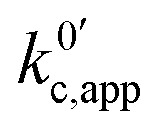
 increase.

The finite element simulations also allow us to easily visualize how the individual cathodic (reduction, negative current) and anodic (oxidation, positive current) peak positions shift with changing [Li^+^] or reaction kinetic parameters. These positions are a proxy for the overpotentials obtained in a galvanostatic experiment, such as a Li–air battery: the higher the shift from a reversible CV shape, the higher overpotentials are expected in a battery configuration. The three different pathways show different behaviours for each of the processes as function of changing [Li^+^]. In the case of pathway 1, the cathodic peak undergoes a small shift towards negative potentials, while the anodic peak is more noticeably pushed towards positive potentials. Mathematically, this arises from the compounded effect of the shift in *E*^0^_app_ (eqn (12)), that moves the average potential between the peaks, with an increase in peak-to-peak separation due to the ET becoming more sluggish. Chemically, these shifts can be rationalized in terms of the Q and Q^−^ concentration: when the [Li^+^] is increased, the vertical equilibria in Scheme 1 are shifted toward the associated species (bottom). This means that the Q and Q^−^ concentrations in the surface of the electrode are always lower than [Q]_T_, which delays their conversion through pathway 1.

For pathways 2 and c, the opposite trend is seen: the cathodic peak suffers a larger shift in potential than the anodic one. In this case, the displacement of the system towards higher association degrees increases the concentration of electroactive species, Q–Li^+^ and Q–Li, making the overall system more reversible. Interestingly, this means that for a RM following pathway 1, its reduction potential does not change drastically with lithium salt concentration, but for one following either pathway 2 or c, the reduction potential will be highly dependent on it.

#### 1.4 Effect of multiple ion association

Now that we have analysed in detail the simplified system, we extend the treatment to allow the RM to associate with multiple Li^+^ ions, as is the case in DMSO and our model system in this work.^15^ By following the same procedure as the one described above, one can determine how the individual standard rate constants *k*^0^_*i*_ change with varying [Li^+^]. The generalized multiple association equations are given by:27
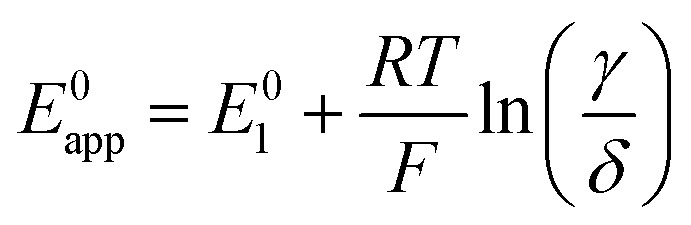
where28
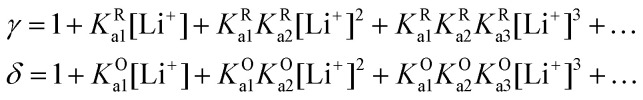
And for the apparent rates of the *i*th electrochemical and concerted processes:29
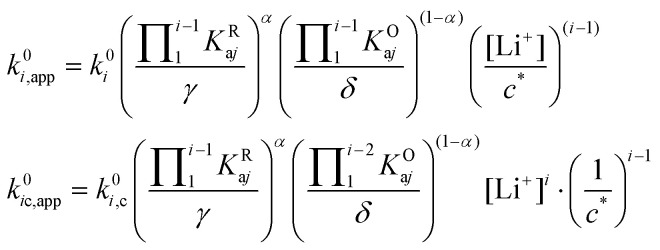
Here, *K*^R^_a*i*_ and *K*^O^_a*i*_ correspond to the monocationic association constants of the reactions QLi_i_^(*i*−1)+^ + Li^+^ ⇌ QLi_(*i*+1)_^*i*+^ and QLi_*i*_^*i*+^ + Li^+^ ⇌ QLi_(*i*+1)_^(*i*+1)+^ respectively, as depicted in the vertical reactions of Scheme 1a. *k*^0^_*i*_ and *k*^0^_*i*,c_ are the standard kinetic ET constants for the monomolecular (horizontal) and concerted bimolecular (diagonal) ET steps depicted in Scheme 1a, respectively, and *k*^0^_*i*,app_ and *k*^0^_*i*c,app_ the apparent standard ET constants for pathway *i* and *i*c when the chemical equilibria is considered.

**Fig. 3:**
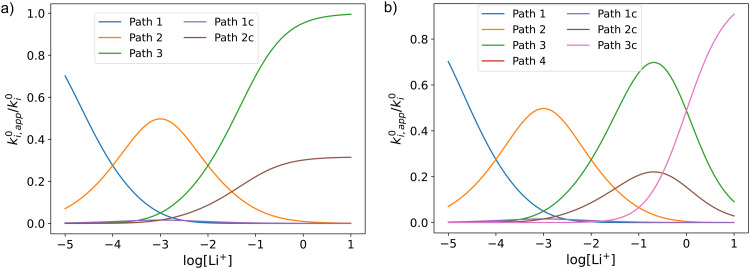
Example of behaviour of the apparent standard rate constant for each of the pathways represented in Scheme 1a with *n* = 2 (a) and *n* = 3 (b). The plots shown in the figures were computed using the parameters *K*^R^_a1_ = 1 × 10^5^, *K*^R^_a2_ = 1 × 10^3^, *K*^O^_a1_ = 1000, *K*^O^_a2_ = 10, and *K*^R^_a3_ = *K*^O^_a3_ = 0 for (a) and *K*^R^_a3_ = *K*^O^_a3_ = 1 for (b). The parameters were chosen so that the behaviour of the different pathways can be seen clearly. The actual positions and magnitudes of the curves’ maxima and plateaus strongly depend on these parameters, but their shapes are characteristic of the different paths.

Fig. 3 shows the evolution of *k*^0^_*i*,app_ with [Li^+^] for the different pathways when the maximum association steps considered correspond to *n* = 3 (Fig. 3a) and *n* = 4 (Fig. 3b), representing the association of Q with two and three Li^+^ ions, respectively. Interestingly, allowing for association with multiple cations makes the pathways’ apparent rate constants non-monotonic in [Li^+^] resulting in different concentration regions where each pathway is dominant. In particular, if *K*^R^_a2_ > *K*^O^_a2_ (which is expected based on electrostatic interactions), pathway 2 shows a maximum at intermediate [Li^+^]. This can be easily understood, by a shift of the equilibria governed by *K*^O^_a2_ and *K*^R^_a2_ to products at high [Li^+^], lowering the available [QLi^+^] and [QLi] to react, hence making path 2 and 1c unfavourable. Interestingly, this suggests that some of the possible pathways the reaction might take could be disentangled by examining experimental CVs as a function of [Li^+^]. Note that pathways 2 and 1c and 3 and 2c have the same functional dependence on [Li^+^], an enlargement of these curves can be seen in Fig. S2 (ESI†). This statement can be generalized to pathways *i* and (*i* + 1)c.

#### 1.5 Experimental application of the theoretical framework to DBBQ-containing DMSO electrolytes

**Fig. 4:**
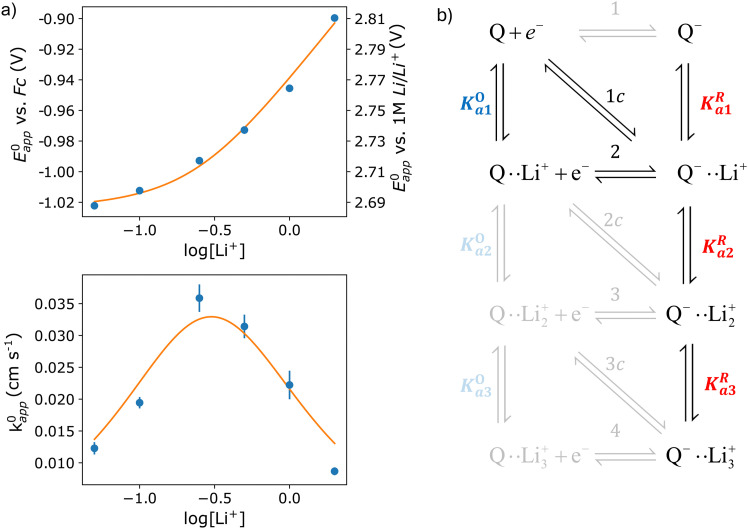
(a) Experimental standard potential, *E*^0^ (top) and kinetic constant, *k*^0^ (bottom) represented as dots compared with the fitting results when assuming the mechanism represented in panel (b). The values derived from the fits are given in Table 1. (b) Schematic of the possible pathways for redox activity of DBBQ in Li^+^ containing DMSO solutions. The greyed-out pathways were not needed to explain the experimental behaviour.

In this section, we describe the application of the previously developed framework to gain insight into DBBQ reduction pathways in Li^+^ containing DMSO electrolytes. Values of *E*^0^_app_ and *k*^0^_app_ as a function of [Li^+^] were obtained by measuring cyclic voltammetry curves at varying LiTFSI concentrations and scan rates. The obtained results for *E*^0^_app_ and *k*^0^_app_ for the electrolytes tested can be observed in Fig. 4a, and an example of the CVs in Fig. S1 in the ESI.† Similar measurements have been previously reported by Bawol *et al.*,^15^ where they measure the evolution of the standard potential of DBBQ reduction in DMSO at different LiClO_4_ concentrations, concluding that DBBQ binds to between two and three Li^+^ ions. Our results extend the concentration regime to one order of magnitude lower (from 0.5 M to approximately 50 mM), making the nonlinearities of the *E*^0^_app_*vs.* log[Li^+^] plot more evident. Moreover, we analyse the evolution of the kinetics with [Li^+^] in the terms of the proposed framework, expanding their analysis and extracting mechanistic information related to the pathways. Since we are working with a wide range of salt concentrations, where density and hence volume changes can have a large impact, the LiTFSI concentrations here are expressed in terms of molality.

The experimental data obtained for *E*^0^_app_ and *k*^0^_app_ were fitted using the mechanism shown in Fig. 4b. Multiple combinations of the possible pathways were tested, including the greyed-out reactions, with constraints placed on the association constants based on expected electrostatic interactions. Namely, the constants were set to follow the order *K*_a1_ > *K*_a2_ > *K*_a3_ for both the oxidized and reduced species, and *K*^R^_a*i*_ > *K*^O^_a*i*_ for constant *i*, with the same number of interactions with Li^+^. We found that only pathways 2 and 1c were necessary to describe the experimental results, and adding more alternative pathways did not improve the fitting significantly, so they were kept inactive for the final fit. The parameters corresponding to the highlighted active pathways were varied to fit the evolution of *E*^0^_app_ and *k*^0^_app_*vs.* the lithium-ion concentration, and the results can be found in Table 1.

**Table 1:** Parameters obtained by fitting experimental *E*^0^_app_ and *k*^0^_app_ to the model described in Fig. 4b. Potentials are reported using ferrocene (Fc) internal reference, and Li/Li^+^ 1 M in LiTFSI as external reference

Parameter	Best fit value
*E* ^0^_1_	−1.0 *vs.* Fc/2.7 *vs.* Li
*E* ^0^_2_	−0.97 *vs.* Fc/2.73 *vs.* Li
*E* ^0^_c_	−0.96 *vs.* Fc/2.74 *vs.* Li
*K* ^R^_a1_	5.4
*K* ^R^_a2_	3.2
*K* ^R^_a3_	3.2
*K* ^O^_a1_	1.9
*k* ^0^_2_ (*K*^O^_a1_)^(1−*α*)^ + *k*^0^_1c_	0.102 cm^4^ mol^−1^ s^−1^

The first thing that becomes clear is that the quinone is highly associated in this system, binding to up to three Li^+^ ions in its reduced form. However, the neutral oxidized form also interacts with Li^+^, something that was not considered in literature before. In fact, our results indicate that, at the typical 1 M concentration, around 65% of the neutral DBBQ is bound to a Li^+^. Since the apparent kinetics of pathways 2 and 1c follow the same functionality with lithium concentration, it is not possible to separate the contributions from each of them with this approach. However, it is clear from the fitting results that pathway 1 and 2c are inherently much slower than 2/1c. *I.e.*, the association of DBBQ with Li^+^ either prior to reduction (1) or *via* a concerted process (2c) result in faster reduction kinetics. Steps 2 and 1c are associated with larger free energy changes than step 1, since *E*^0^_1_ < *E*^0^_2_ < *E*^0^_c_ (eqn (7) and (8) still hold). In both cases, an activated complex involving one Q molecule and 1 Li^+^ ion is formed. It can then be speculated that the association of the quinone with a Li^+^ ion modifies the electronic structure of the quinone ring in such a way that it lowers the energy of the products and activated complex, thus making these pathways kinetically predominant, *i.e*, they have a higher *k*^0^.

#### 1.6 Effect of competing equilibria: supporting salt and solvation strength

We now move on to broaden our theoretical model to other phenomena that can play an important role in the ET mechanism. Particularly, in low donor number solvents, such as the glyme family, it is known that the supporting salts undergo significant association with the lithium ion, either as contact ion pairs or as solvent separated ion pairs, which will then affect the availability of the lithium ion to bind to the RM.^35,36^ For example, it has been shown that in diglyme (DG) and monoglyme (DME), the LiTFSI association constant can be up to 10^6^.^35^ Other widely used salts, such as lithium trifluoromethanesulfonate (LiTf), LiNO_3_, LiPF_6_ and LiClO_4_ have also shown significant degrees of ionic association in glymes.^37–40^ Furthermore, additives or impurities such as water have also been shown to affect the RM reduction processes *via* similar processes, complexing to the Li^+^ ions^25^ and competing with the quinone for the lithium ions present in solution, decreasing the available free lithium concentration dramatically.

In this section, we consider the thermodynamics and kinetics of the RM reaction network discussed previously, with the addition that the Li^+^ ion may also interact with its counterion, X^−^. We limit our analysis to the case where Q binds to only 1 Li^+^ ion, as in Scheme 1b (*i.e. n* = 1) for simplicity. The extension to higher *n* values is trivial, and the effect on the system is analogous. In this case, mass balance eqn (11) is replaced by:30
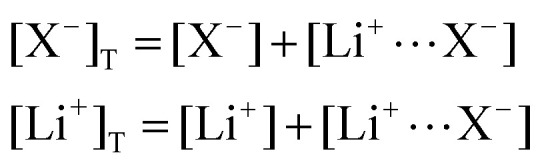
where X^−^ represents the counteranion of the lithium salt used in the electrolyte and the subindex “T” refers to the total analytical concentration of a species. We have assumed that the concentration of the RM is much smaller than the supporting salt concentration (which is usually the case in Li–O_2_ batteries). Under these circumstances, eqn (12) for the apparent standard potential still holds, but the free lithium concentration is now much lower than the total lithium concentration, and its value can be calculated as31
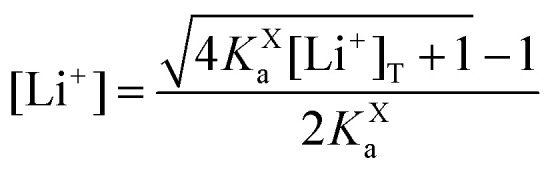
from mass balance eqn (30) and where *K*^X^_a_ represents the association constant of the lithium salt, corresponding to the reaction Li^+^ + X^−^ ⇌ LiX. To illustrate, *K*^X^_a_ is 5 × 10^4^ in the case of LiTFSI in DME.^35^ Therefore, the free lithium concentration in a 0.1 M solution is 0.0014 M, while in a 1 M solution it is 0.0045 M, only a threefold increase for tenfold change in the total concentration. The presence of a strongly associated counteranion acts as a Li^+^ buffer making the free Li^+^ concentration much smaller and approximately constant.

**Fig. 5:**
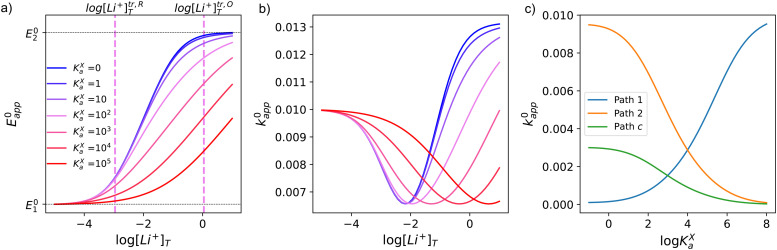
Variation of apparent standard potential (a) and total rate constant (b) with supporting salt concentration for different values of supporting salt association constant, *K*^X^_a_ varying between 0 and 10^5^. Vertical pink dashed lines represent the transition concentrations for the case of *K*^X^_a_ = 100, corresponding to the pink curve, as an example. (c) Variation of the contribution of each pathway with change in *K*^X^_a_. The plots in the figures were computed using *K*^O^_a_ = 10, *K*^R^_a_ = 1000, *k*^0^_1_ = *k*^0^_2_ = *k*^0^_c_ = 0.01 for (a) and (b), and [Li^+^]_T_ = 1 M for (c).

The dependence of ET parameters on association strength of the supporting salt is shown in Fig. 5. The apparent potential plateaux corresponding to pure pathway 1 and pure pathway 2, and the crossover region between them, now depend on *K*^X^_a_ and are governed by eqn (12) and (31). The transition from *E*^0^_1_ to *E*^0^_2_ no longer follows a straight line with slope *RT*/*F*, but it slows down to a slope of *RT*/2*F* when *K*^X^_a_ is sufficiently high. The transition between the two extreme regimes is shifted to higher [Li^+^], the value of this crossover concentration is stated in eqn (S4) of the ESI.†

Fig. 5 shows the effect of varying *K*^X^_a_ on the thermodynamics and kinetics of the system. The full equations describing these phenomena can be found in eqn (S2)–(S4) (in the ESI†). As expected because of the small amount of free lithium in highly associated solutions, all changes with increasing [Li^+^] are delayed and happen in a less abrupt way.

Interestingly, sufficiently high competition for the lithium ions can affect the dominant pathway taken by the redox reaction. This becomes evident when we analyse how each of the contributions of *k*^0^_1,app_, *k*^0^_2,app_ and *k*^0^_c,app_ to the total *k*^0^_app_ behave as a function of *K*^X^_a_ at a given [Li^+^], in Fig. 5(c). We can see how higher association of the lithium salt promotes pathway 1 while slowing down pathways 2 and c. The overall mechanism is, in the end, a competition between the association of the quinone and of the supporting counteranions with lithium. We note that this effect is not important in DMSO based electrolytes due to the low ionic association of typical Li–air battery salts in this solvent, however it is expected to be relevant in glyme based electrolytes.

The “free” lithium concentration can also change due to strong interactions with solvent molecules (particularly in systems with high lithium solvation energies). For example, in the case of glymes, it has been shown that Li^+^ forms complexes with the solvent molecules that stabilize the Li^+^–glyme adducts with respect to free Li^+^.^35,41–45^ The present system can be treated analogously, with the only difference that the solvent molecules are in large excess with respect to the lithium ions. The effect of solvation strength, *i.e.* Δ*G*^0^ for the solvation reaction, on the standard apparent potential and rate constants is depicted in Fig. S3 in the ESI.† Qualitatively, the Li^+^ solvation has a similar effect to that of Li^+^ association with the counteranion, and experimentally a combination of both effects is likely seen. Extending the analysis of this section to *n* > 1 is straightforward by combining eqn (27)–(29) with eqn (31).

Overall, in this section we have shown how the strong interaction of quinone with Li^+^ ions affects the reduction mechanism, making the reaction rate and apparent potential depend on lithium concentration, and on solvent and supporting salt identities. We have shown that for a given RM-Li^+^ interaction strength (*K*^O^_a_ and *K*^R^_a_), the free Li^+^ concentration can tune the kinetics of the system. We also discussed the effect of parallel equilibria, showing that the defining parameter is the free [Li^+^], which can be influenced by ionic association of Li^+^ with the counteranion and by its interaction with solvent molecules. Taking these results together, we conclude that to optimise electrolyte composition for minimum overpotential one must first establish the free-lithium concentration that maximises the apparent reduction rate constant in the absence of supporting salt, and then tune the supporting salt concentration to achieve that concentration. Kinetic models such as the framework presented here offer the possibility of determining these target concentrations with a reduced number of experiments, especially if some of the parameters required can ultimately be predicted computationally or else inferred from other solvent systems.

### 2. Effect of ionic association in catalysis of the ORR

At this point we have built a comprehensive framework to describe the electrochemical behaviour of RM systems, particularly DBBQ. We now consider whether we can we use the apparent parameters obtained in the previous section to help explain the behaviour of the Li–O_2_ cathode reactions, involving the catalytic processes of DBBQ reacting with O_2_. Since there are no systematic studies and understanding of the CV shapes of mediated ORR in Li–air electrolytes, the goal of this section is to provide a guide that can help rationalize experimental results as a step towards a unified mechanism of the DBBQ mediated ORR.

We start by describing the simplest case, described with eqn (32), where O is the catalyst (in our case DBBQ) and R its reduced form, S the substrate (in our case oxygen, O_2_), and P the product (in our case Li_2_O_2_). It is well established in literature that homogeneous electrocatalytic processes of this type can be characterised by the shape of their cyclic voltammogram. This shape depends on the analytic concentration of O, *c*_O_, the analytic concentration of S, *c*_S_, the scan rate, *υ*, and the rate constant for the catalytic step in eqn (32b), *k*_Cat_. The actual mechanism of DBBQ catalysis is much more complex, but qualitatively similar CV shapes are expected even for more complicated mechanisms.^46–48^

32a O + e^−^ ⇌ R

32b
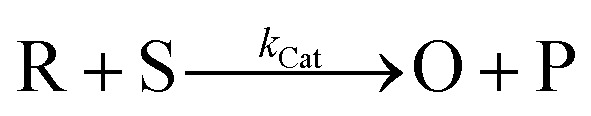


**Fig. 6:**
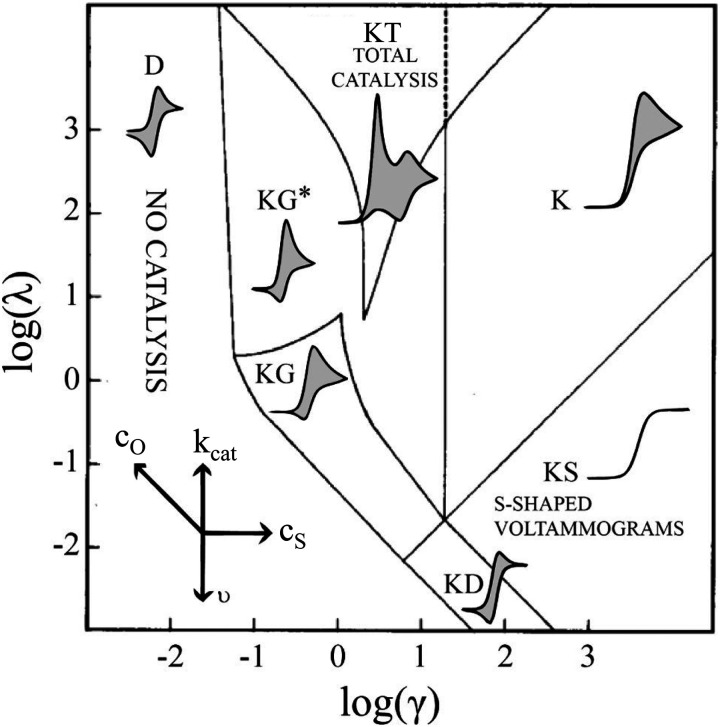
Diagram of the catalytic zones for simulated CVs of one electron reduction of substrate S *via* a redox mediator, O. The kinetic parameter 
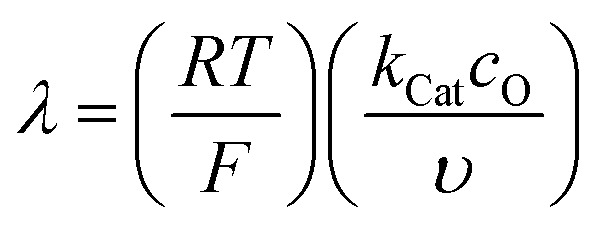
 and the excess factor 
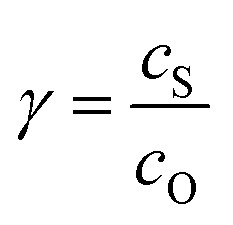
 represent the competition between catalytic rate and scan rate, and the initial electrolyte composition, respectively. The CV waveforms follow the convention of negative potentials to the right and cathodic current upward. Scans are started from positive potentials. Reprinted with permission from ref. 39. Copyright 2014 American Chemical Society.

In short, 6 zones can be identified for different characteristic CV shapes, as described by Rountree *et al.* in their figure reproduced in Fig. 6: D (no catalysis, CV shape observed is equal to the redox couple O/R). KS (pure kinetic conditions, no substrate consumption, S shaped), K (pure kinetic conditions, substrate consumption, large catalytic reduction peak with no oxidation peak), KT (“total catalysis”, pure kinetic conditions, substrate consumption: two reduction peaks), KD (mixed kinetic-diffusion, no substrate consumption, elongated shape), KG (mixed kinetic-diffusion, with substrate consumption: asymmetric peak heights, larger reduction than oxidation peak).^46–48^

When we move on to DBBQ's signature CVs, even though its catalytic mechanism is more complex than this idealised case, similar shapes of CV can be identified in the literature.^16^ These shapes depend not only on the concentration of DBBQ and O_2_, but also on [Li^+^] and solvent choice. For example, for 10 mM DBBQ in 1 M LiTFSI TEGDME electrolyte, a KD type shape has been observed, and in 1 M LiTFSI DME electrolyte a KG type CV has been found, both at 100 mV s^−1^ scan rate.^16^ These observations suggest that DME promotes a higher observed *k*_Cat_, an effect that merits further explanation.^16^

Since there is no clear consensus regarding the mechanism of DBBQ-mediated ORR in different electrolytes, for the purposes of this section, we model the reaction going through LiO_2_ intermediate, *i.e.*eqn (3) and (6), to exemplify the compounded effects of ET and catalytic kinetics in CVs. Further arguments supporting the selection of these reaction mechanisms can also be found in the ESI.† Qualitatively, similar effects can be expected in the case of the other mechanisms. Quantitative differences in the observed catalytic constant depending on [Li^+^] are described in eqn (S8)–(S10) (ESI†).

**Fig. 7:**
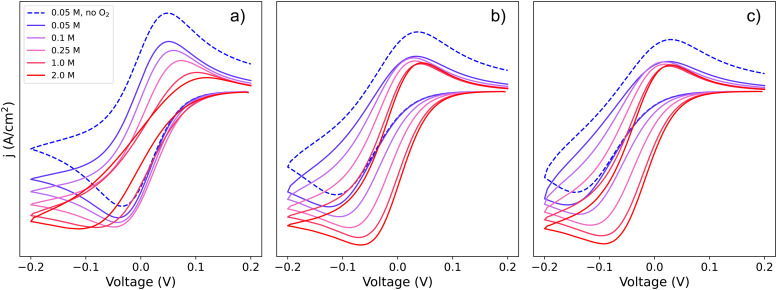
Simulated cyclic voltammograms for each of the DBBQ reduction pathways depicted in Scheme 2, pathways (a) 1, (b) 2, and (c) c, followed by reaction of DBBQ with oxygen according to eqn (3) and (6). The three plots are computed using *K*^O^_a_ = 2, *K*^R^_a_ = 10, *k*^0^_1_ = *k*^0^_2_ = *k*^0^_3_ = 0.01 cm s^−1^, *k*_4_ = 50 and *k*_5_ = 10^10^, and a scan rate of 50 mV s^−1^ at different Li^+^ concentrations as stated in the legend. A simulated CV in the absence of O_2_ and 0.05 M Li^+^ is also shown for comparison, depicted by the blue dashed lines.

Since Li^+^ takes part in both the electrochemical conversion of quinone species (analysed in Section 1.1, Scheme 1) and also the catalytic reactions described in eqn (3) and (6), increasing the [Li^+^] results in the evolution of the shape of the CV shape *via* two superimposed effects. The first effect is related to the change in kinetics of the ET described in Section 1.1, which leads to a modification of the peak-to-peak separation related to the reversibility of ET. The second effect is a modification of the overall observed catalytic constant, which results in the ratio of the peak currents differing from 1 and changing with [Li^+^]; curves with evolving shapes correspond to different catalytic regimes. Fig. 7 shows simulated cyclic voltammograms representing the pathways 1, 2 and 1c discussed in the previous section represented in the simplified Scheme 1b, plus the catalytic reactions (3) and (6) involving O_2_ association and LiO_2_ formation, respectively, at different Li^+^ concentrations (with *n* = 1 for simplicity).

Two effects can be seen in the shape of the CVs with increasing Li^+^ concentration: a change in overpotentials due to the dependence of *k*^0^_app,i_ on [Li^+^], as discussed in Section 1, leads to an increase of peak-to-peak separation in the case of pathway 1 and a decrease for pathways 2 and c. The second effect is a noticeable increase in the catalytic activity when increasing Li^+^ concentration in all three cases. The latter effect leads to a change in the CV shape from region D (no catalysis), to KD (mixed kinetic-diffusion, no substrate consumption), and eventually, for pathway 1, to KG (mixed kinetic-diffusion, with substrate consumption).

The effect on the observed catalytic constant is the same regardless of the pathway for quinone reduction, and depends only on the degree of association and consequent QLi_*n*_ concentration (*i.e.*, [Li^+^] and *K*^R^_a_). However, the efficiency of the overall catalytic process depends on the pathway: for pathways 2 and c, the overpotential for the catalytic process decreases with increasing [Li^+^], as evidenced by the shift of the reduction peak and onset potential to higher voltages. In contrast, for pathway 1, the onset and peak potentials are shifted to more negative values as [Li^+^] increases, which can be easily observed in the highly-distorted shape of the 2 M [Li^+^] curve in Fig. 7a. In other words, as Li^+^ concentration increases, the overpotential also increases, making the catalytic process less efficient (higher overpotentials are needed to access a the same current).

Returning to our theoretical framework, an overall positive effect is seen when both the ET *k*^0^_app_ and the observed *k*_cat_ increase: lower overpotentials and a rise in catalytic rates accompany increasing [Li^+^] (pathways 2 and c), but when *k*^0^_app_ decreases, a balance between the increasing observed *k*_cat_ and decreasing *k*^0^_app_ must be achieved to get an optimized system with low overpotentials and fast catalysis, which is the case of pathway 1.

Analogous analyses can be performed for the case where the quinone associates with more than one Li^+^ ion, as in the example case of DMSO in this work. Previous work has shown that ionic association is important in the catalysis of ORR,^15^ however, it is still unclear which quinone species is the active one (*i.e.*, what the value of *n* in QLi_*n*_ is in eqn (4)–(6)). Our framework will permit including an accurate description of DBBQ association into the kinetic interpretation of experimental results, which has the potential to narrow down the underlying active species. Future work will be devoted to a systematic exploration of the kinetics under different conditions.

## Conclusions

A theoretical framework is presented to understand the effect of the interactions between redox mediators for Li–O_2_ batteries and other electrolyte components on the kinetics and thermodynamics of electron transfer, and subsequent effect on catalytic efficiency. We focus on the effect of Li^+^ ion association, and use DBBQ as an example where these effects can be observed *via* the CV curves.

We provide analytical equations to understand how the observed reduction potential and electron transfer rate constants of an electrochemically active species evolve with Li^+^ concentration, and show how these predictions can be used to interpret experimental data and hence discriminate between different mechanisms of redox activity. By comparing our theoretical predictions with CV experiments, we show that DBBQ binds to up to 3 Li^+^ in DMSO solutions in its monoanionic reduced state and 1 Li^+^ ion in its neutral form. We also show that our predictions can explain the evolution of potential and electron transfer rate constant in these solutions, reproducing a maximum in the observed standard rate constant value, *k*^0^_app_, for DBBQ redox reactions at around 0.25 M LiTFSI. We also briefly discuss the effect of further parallel equilibria, showing that the important parameter is the free Li^+^ concentration, which can be affected by ionic association of Li^+^ with the supporting salt counteranion or its interaction with solvent molecules. This approach can also be extended to understand the effect of additives or impurities such as water, which have been previously shown to affect the redox behaviour in a similar way.

Finally, we show how the catalysis of ORR using DBBQ is affected by the Li^+^ concentration, considering its effect on the electron transfer and catalytic kinetics, and showing example cyclic voltammograms which are seldom analyzed in detail. We show how the shape of CVs where ORR is catalyzed by DBBQ can change significantly just by varying [Li^+^], shifting between different catalytic regimes, and we predict how these signature shapes will evolve depending on quinone redox pathways.

Our analysis is important in three ways: first, it gives predictive power to translate the electrochemical behaviour of associated RM from one set of conditions to another (*e.g.*, changing the solvent or the supporting salt) based on physically meaningful parameters, such as the association constants or solvation energies. Second, it opens up new dimensions available to rationally improve the kinetics, and hence overpotentials and rate performance, of the quinone reactions in relevant electrolytes. Finally, it simplifies the system by providing apparent parameters that can be used further to understand the catalytic reaction of DBBQ with O_2_, as demonstrated in Section 2, providing an accurate but simple way to incorporate the complex electrochemical processes of the redox couple. The strength of our theoretical framework lies in its flexibility to be adapted to the use of different redox mediators and interactions with electrolyte components. Future work will focus on building a generalized understanding of the DBBQ behaviour in Li^+^ containing solutions by applying this framework to different electrolyte compositions, ultimately extending it to systems containing O_2_.

## Experimental and simulation details

The experimental data was collected by running cyclic voltammetry of solutions containing 5 mM DBBQ at different LiTFSI concentrations in DMSO solvent, at varying scan rates between 20 mV s^−1^ and 10 V s^−1^. The solutions were prepared by weighing the corresponding amounts of DBBQ (Sigma Aldrich, 99% purity), LiTFSI (Sigma Aldrich, 99.95% purity) and anhydrous DMSO (Sigma Aldrich, 99.9% purity) in an Ar filled glovebox. Prior to use, DBBQ was further purified by recrystallization in methanol: approximately 5 g of DBBQ were recrystalized in methanol, decanted and dried under vacuum. This process was done twice and yielded yellow needle-like crystals. The solutions were prepared on the same day to the electrochemical measurements to avoid degradation of DBBQ with light, since old light-exposed solutions were observed to change colour with time. To be able to convert between different concentration units, the density of each solution was measured three times by weighing 1 ml of solution from a pipette.

All electrochemical experiments were recorded inside an Ar-filled glovebox using a PalmSense 4 potentiostat. A reference electrode composed of a silver wire coated in Li_1.5_Mn_2_O_4_ inside a fritted compartment with 1 M LiTFSI/tetraglyme electrolyte as described elsewhere,^49^ a Pt wire counter electrode, and a 3 mm diameter glassy carbon working electrode were used. There were three repeats of each set of measurements including all the scan rates in each solution. Electrochemical Impedance Spectroscopy (EIS) was recorded before and after each set of measurements with a voltage amplitude of 10 mV and a frequency range of 5 to 50 000 Hz. The obtained Nyquist plot was fitted using the PSTrace 5.8 software to a Randles circuit to obtain the resistance and capacitance of the solution.

Prior to running any electrochemical measurement, the OCV *vs.* Li/Li^+^ couple was measured by submerging a Li strip into the electrolyte for a few seconds, and after all the CV and EIS experiments were performed, a small amount of ferrocene was added to the solution and a new CV was recorded. The average voltage between the oxidation and reduction peaks of Fc/Fc^+^ couple was then used as an internal reference.

The voltammograms were fitted with the commercially available software DigiElch^34^ to a single electron transfer event (as shown in Scheme 1c), represented by the parameters *E*^0^, *k*^0^ and *α*. The resistance and capacitance of the solutions obtained by EIS were provided to the simulation software to account for *iR* drop and capacitive currents. The diffusion coefficients of the oxidized and reduced species (*D*^O^ and *D*^R^) were assumed to be equal due to the small changes expected in the size of the molecules. The value of *α* was kept as 0.5, since allowing for its variation didn’t present any further improvement of the fits, and *E*^0^ and *k*^0^ were systematically varied to achieve the best fit of the experimental voltammograms over the measured range of scan rates for each concentration.

The evolution of the experimentally obtained *E*^0^_app_ and *k*^0^_app_ were fitted to eqn (27) and (28). Each of the possible pathways was added to the simulated values one at a time, until no significant further improvement of the theoretical curve was seen. Boundary conditions were imposed for the fitting based on expected values of the constants: *k*^0^_*i*_, *k*^0^_*i*c_, *K*^R^_a*i*_ and *K*^O^_a*i*_ > 0, *K*^R^_a*i*_ > *K*^O^_a*i*_, *K*^R^_a*i*_ < *K*^R^_a(*i*+1)_, *K*^O^_a*i*_ < *K*^O^_a(*i*+1)_. This ensures each uptake of a Li^+^ is less favourable than the previous one, and that less positive charged species have a higher tendency to bind to Li^+^ than more positively charged ones.

## Data availability

The data supporting this article have been included as part of the ESI.†

## Conflicts of interest

There are no conflicts to declare.

## Supplementary Material

CP-026-D4CP01488J-s001
