# Supplementary material for: The effect of ionic association on the electrochemistry of redox mediators for Li–O2 batteries: developing a theoretical framework
Source: Phys Chem Chem Phys. 2024 Jul 1;26(33):22134–48. doi: 10.1039/d4cp01488j (PMC11310830; doi:10.1039/d4cp01488j)
Supplement: CP-026-D4CP01488J-s001 [file CP-026-D4CP01488J-s001.pdf]

Electronic Supplementary Material (ESI) for Physical Chemistry Chemical Physics.
This journal is © the Owner Societies 2024

Supporting Information for: The Effect of Ionic Association on the Electrochemistry of Redox Mediators for Li-O_2 Batteries: Developing a Theoretical Framework

Gabriela Horwitz,[a] Vera Kunz,[a] Samuel Niblett,[a] and Clare P. Grey*[a]

[a] Yusuf Hamied Department of Chemistry, University of Cambridge.
Lensfield Rd, Cambridge CB2 1EW, United Kingdom

[b] The Faraday Institution,
Quad One, Harwell Campus, Becquerel Ave, Didcot OX11 0RA, United Kingdom

Additivity of rates:

If we define the total rate of the reduction reaction as the change in the total concentration of oxidized quinone species though any of the three pathways described before, we can write that

$$\begin{aligned} v_{tot}^{red} = -\frac{d[Q]_T}{dt} &= k_1^{red}[Q] + k_2^{red}[Q \cdot\cdot Li^+] + k_3^{red}[Q][Li^+] \\ &= k_{1,app}^{red}[Q]_T + k_{2,app}^{red}[Q]_T + k_{3,app}^{red'}[Q]_T \\ &= \left(k_{1,app}^{0} + k_{2,app}^{0} + k_{3,app}^{0'}\right)\exp\left[-\frac{\alpha F}{RT}\left(E - E_{app}^{0}\right)\right][Q]_T \end{aligned} \quad (S1)$$

Which depicts that the total rate law can be expressed as a function of a new rate constant composed of the addition of the three pathway's rate constants.

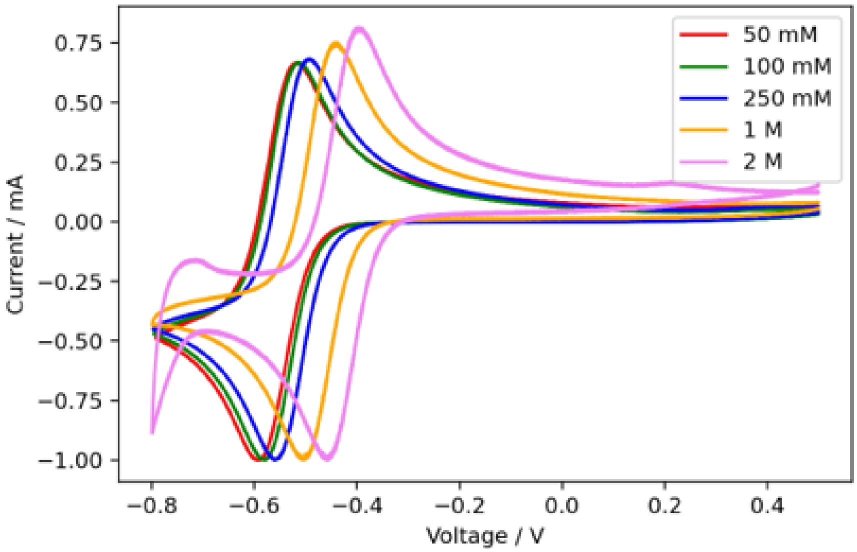

Figure S1: Example cyclic voltammograms of solutions of 5mM DBBQ in varying LiTFSI concentrations in DMSO. The currents are normalized to the cathodic peak for an easier visual comparison, and the voltage referenced to $Li_{1.5}Mn_2O_4$ in 1M LiTFSI/tetraglyme solution.

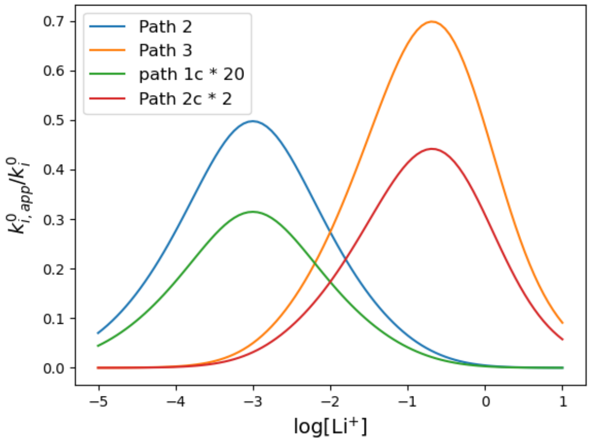

Figure S2: Enlargement of Figure 3 for the pathways that show the same functional behaviour with [Li^+], for n=3. The plots shown in the figures were computed using the parameters $K_{a1}{}^{R}$=1x10^5, $K_{a2}{}^{R}$=1x10^3, $K_{a1}{}^{O}$=1000, $K_{a2}{}^{O}$=10, and $K_{a3}{}^{R}$= $K_{a3}{}^{O}$=1. Pathways 1c and 2c are scaled to allow for an easier visual comparison.

Effect for supporting salt association

From equation (25), the free lithium concentration can be introduced in the apparent standard potential expression, to obtain a new expression of the apparent rate constant in a system with

parallel association equilibria with the supporting salt counteranion. Here is the derivation for pathway 1, analogous treatment can be done for pathways 2 and *c* to obtain the total rate changes observed in Figure 5.

Starting from equation (8) and considering the free [Li⁺] given by equation (26), the apparent standard potential can be written as:

$$E^0_{app} = E^0_1 + \frac{RT}{F}\ln\left(\frac{1+K_a^R[Li^+]}{1+K_a^O[Li^+]}\right)$$
$$E^0_{app} = E^0_1 + \frac{RT}{F}\ln\left(\frac{1+\frac{K_a^R}{2K_a^{Sup}}\left(\sqrt{4K_a^{Sup}[Li^+]_T+1}-1\right)}{1+\frac{K_a^O}{2K_a^{Sup}}\left(\sqrt{4K_a^{Sup}[Li^+]_T+1}-1\right)}\right) \quad \text{(S2)}$$

And the evolution of k01,app, from equation (15) can be rewritten as:

$$k^0_{1,app} = k^0_1\left(1+K_a^R[Li^+]\right)^{-\alpha}\left(1+K_a^O[Li^+]\right)^{\alpha-1}$$
$$k^0_{1,app} = k^0_1\left(1+\frac{K_a^R}{2K_a^{Sup}}\left(\sqrt{4K_a^{Sup}[Li^+]_T+1}-1\right)\right)^{-\alpha}\left(1+\frac{K_a^O}{2K_a^{Sup}}\left(\sqrt{4K_a^{Sup}[Li^+]_T+1}-1\right)\right) \quad \text{(S3)}$$

The transition concentrations are given by the condition of $\frac{K_a^R}{2K_a^{Sup}}\left(\sqrt{4K_a^{Sup}[Li^+]_T^{tr,R}+1}-1\right)$=1 and $\frac{K_a^O}{2K_a^{Sup}}\left(\sqrt{4K_a^{Sup}[Li^+]_T^{tr,O}+1}-1\right)$=1 , concentrations of Li^+ at which the relations between associated and free quinone species are equal, *i.e.* [QLi]/[Q^-] = 1 and [QLi^+]/[Q] = 1, respectively.

$$[Li^+]_T^{tr,R} = \frac{1}{K_a^R}\left(\frac{K_a^X}{K_a^R}+\frac{1}{2}\right)$$
$$[Li^+]_T^{tr,O} = \frac{1}{K_a^O}\left(\frac{K_a^X}{K_a^O}+\frac{1}{2}\right) \quad \text{(S4)}$$

Effect of solvation energy

The complexation of Li^+ ions by solvent molecules can be modelled by the following reaction, characterized by a formation constant of the complex related to the free energy stabilization of Li^+ in solution K_a^{sv}.

$$Li^{+} + n\ Sv \rightleftharpoons LiSv_{n} \qquad (S5)$$

If we made the assumption that the activity of the solvent is not too different from the unity, under the assumption that the solvent molecules taking part on the interaction are negligible with respect to the total number of solvent molecules (which is not strictly valid at around 1 M, but it allows for a qualitative description), the expression for free lithium concentration simplifies to equation (S3) for the free lithium concentration, which produces a simple shift in the concentration axis

$$[Li^{+}] = \frac{[Li^{+}]_{T}}{K_{a}^{sv} + 1} \qquad (S6)$$

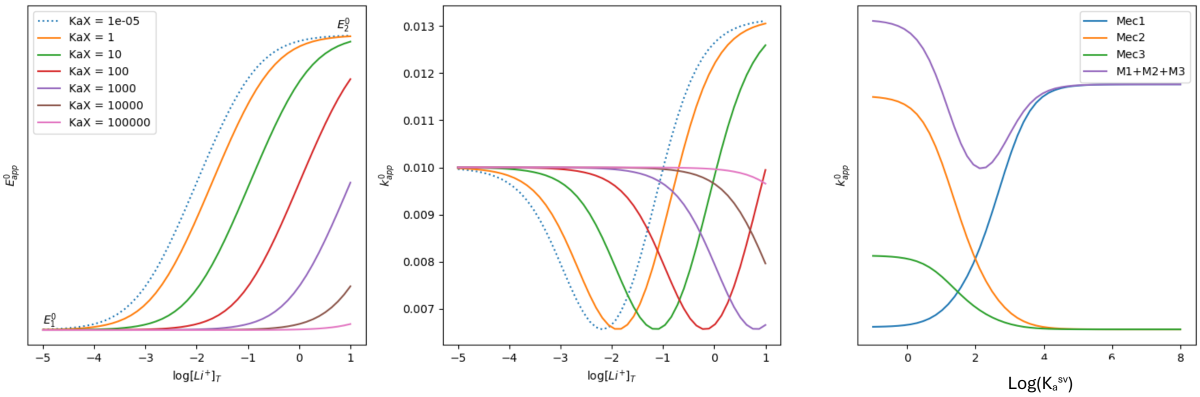

Figure S3: Effect of solvation energy in potential, rate constant and pathways of the apparent simplified mechanism.**Steady state equations for ORR catalysis**

A simplified catalytic mechanism can be described by (assuming n=1, see main text):

$$2Li^{+} \cdot\cdot Q^{-} + O_{2} \xrightarrow{k_{app}^{Cat}} Li_{2}O_{2} \quad 2Q \quad k_{app}^{Cat} \qquad (S7)$$

Under the assumptions described above, and the premise of a steady state concentration of the intermediate species, we can express the rate law and apparent catalytic rate constant, k_{app}^{Cat}, for each of the mechanisms described in the main text as

$$k_{Cat}^{obs} = k_{Cat}^{*}\left(\frac{1}{1 + 1/(K_{a}^{R}[Li^{+}])}\right)^{2}$$
$$rate = k_{Cat}^{obs}[Q]_{T}^{2}[O_{2}]^{2} \qquad (S8)$$

for mechanism in equation (30),

$$k_{Cat}^{obs} = \frac{k_{Cat}^{*}}{2}\left(\frac{1}{1 + 1/(K_{a}^{R}[Li^{+}])}\right)^{2}$$
$$rate = k_{Cat}^{obs}[Q]_{T}^{2}[O_{2}] \qquad (S9)$$

for mechanism in equation (31), and

$$k_{Cat}^{obs} = \frac{k_{Cat}^{*}}{1 + 1/(K_a^R[Li^+])} \quad \text{(S10)}$$
$$rate = k_{Cat}^{obs}[Q]_T[O_2]$$

for mechanism in equations (32). k^*_{cat}, in all three cases corresponds to the rate constant under a vast excess of Li^+ concentration compared to catalysis products, Li_2O_2 and LiO_2, *i. e.* at very fast scan rates or the beginning of the scan in cyclic voltammetry, and at low capacities or very slow discharge rates in battery configuration.

Since it has been shown that DBBQ monoanion needs to be bound to a metal ion to effectively react with O_2, in this case bound to Li^+, the apparent catalytic rate depends on the Li^+ concentration and the K_a^R. It is important to note that the $[Li^+]$ refers to the free lithium, which will change as the reaction evolves until a steady state is established.
In all three catalytic pathways, the lithium concentration has qualitatively the same effect, however, accurate determination of the variation of k_{cat}^{obs} could help dilucidated which is the preferred pathway. In a similar manner, determining the rate variation when using different concentrations of quinone and oxygen can help determine the reaction order of these species and consequently the pathway. Even when this experiments are not straightforward, we can use these proposed mechanism to simulate qualitative changes in cyclic voltammetries, which are seldom analyzed in detail (see main text).

Discussion on the mechanism of ORR catalysis of DBBQ

The different proposed mechanisms of the reaction of DBBQ with oxygen leading to Li_2O_2 are stated in equations (3) to (6) from the main text, and are copied here for the reader's convenience

$$Q^- \cdot\cdot Li_n^+ + O_2 \underset{k_{-1}}{\overset{k_1}{\rightleftharpoons}} Li_n^+ \cdot\cdot Q^- \cdot\cdot O_2 \quad (3)$$

$$2Li_n^+ \cdot\cdot Q^- \cdot\cdot O_2 \xrightarrow{k_2} Li_2O_2 + O_2 + 2\,Q \;\; Li_{n-1}^+ \quad (4)$$

$$Li_n^+ \cdot\cdot Q^- \cdot\cdot O_2 + Q^- \cdot\cdot Li_n^+ \xrightarrow{k_3} Li_2O_2 + 2Q \;\; Li_{n-1}^+ \quad (5)$$

$$Li_n^+ \cdot\cdot Q^- \cdot\cdot O_2 \xrightarrow{k_4} LiO_2 + Q \;\; Li_{n-1}^+ \quad (6.a)$$
$$2LiO_2 \xrightarrow{k_5} Li_2O_2 + O_2 \quad (6.b)$$

It is reasonable to expect the rate of equations (4) and (5) to be slower than that of (3) given the higher activation energy required for electron tunnelling necessary for a redox process in comparison with a mere association reaction. Reactions (4) and (5) are likely also slower than (6.a) since the former require two quinone-containing particles to find each other in solutions that are usually quite diluted (around 10 mM). More evidence supporting the mechanism through reactions (6) is the negligible or very small reduction in the amount of degradation products found in Li-O_2 batteries run with DBBQ-containing electrolytes in DME and TEGDME compared with batteries run without DBBQ.[1,2] The only report showing a significant decrease of side reactions when using DBBQ is in the study using 0.5M $LiClO_4$ / TEGDME electrolyte by Bawol et al.[3] It

is difficult to compare the source of this difference given their distinct setup using an Au electrode and $LiClO_4$ as supporting salt, and the voltage range of their CVs, that go up to lower potentials accessing the second reduction of DBBQ. Hence in our analysis we model the reaction going through the mechanism depicted by equations (6), while a fast equilibrium is established by reaction (3).

1. Liu, T. *et al.* The Effect of Water on Quinone Redox Mediators in Nonaqueous Li-O2 Batteries. *J Am Chem Soc* **140**, 1428–1437 (2018).

2. Gao, X., Chen, Y., Johnson, L. & Bruce, P. G. Promoting solution phase discharge in Li-O2 batteries containing weakly solvating electrolyte solutions. *Nat Mater* **15**, 882–888 (2016).

3. Bawol, P. P., Thimm, J. H. & Baltruschat, H. Unraveling the Mechanism of the Solution-Mediated Oxygen Reduction in Metal-O2 Batteries: The Importance of Ion Association. *ChemElectroChem* **6**, 6038–6049 (2019).
